# Long noncoding RNA POU3F3 enhances cancer cell proliferation, migration and invasion in non-small cell lung cancer (adenocarcinoma) by downregulating microRNA-30d-5p

**DOI:** 10.1186/s12890-020-01218-3

**Published:** 2020-07-02

**Authors:** Qigang Zeng, Yong Dai, Chenxia Duan, Rong Zeng, Qingxiang Zeng, Chengong Wei

**Affiliations:** 1 Department of Respiratory Medicine, Guangdong Provincial Hospital of Integrated Traditional Chinese and Western Medicine, No. 16 Nanwu Road, Guicheng, Nanhai District, Foshan City, Guangdong Province 528200 P.R. China; 2 Clinic of Integrated Traditional & Western Medicine, Shenzhen Longgang District People’s Hospital, Shenzhen City, 518172 P.R. China

**Keywords:** Non-small cell lung cancer, lncRNA POU3F3, microRNA-30d-5p, Cell proliferation

## Abstract

**Background:**

Long noncoding RNA POU class 3 homeobox 3 (POU3F3) is upregulated in esophageal squamous-cell carcinomas. The present study aimed to investigate the role of POU3F3 in non-small cell lung cancer (NSCLC).

**Methods:**

A total of 80 patients with NSCLC (adenocarcinoma) admitted by Guangdong Provincial Hospital of Integrated Traditional Chinese and Western Medicine between May 2016 and May 2018 were enrolled in this study. All patients were diagnosed by histopathological approaches. Expression levels of POU3F3 and microRNA-30d-5p (miR-30d-5p) in cancer and non-tumor tissues from these NSCLC patients were determined by qRT-PCR. Cell transfections were performed to assess interactions between miR-30d-5p and POU3F3. Cell proliferation, Transwell migration and invasion assays were performed to investigate the role of miR-30d-5p and POU3F3 in the regulation of cell proliferation, migration and invasion.

**Results:**

POU3F3 was upregulated, while miR-30d-5p was downregulated in cancer tissues than in adjacent healthy tissues of NSCLC patients. Correlation analysis showed that expression levels of POU3F3 and miR-30d-5p were inversely correlated in tumor tissues. Overexpression of miR-30d-5p did not affect the expression of POU3F3, while overexpression of POU3F3 resulted in the suppression of miR-30d-5p in NSCLC cell lines. Overexpression of POU3F3 mediated enhanced proliferation, migration and invasion of NSCLC cells. In addition, overexpression of miR-30d-5p played an opposite role and attenuated the effects of overexpressing POU3F3 on cancer cell proliferation, migration and invasion.

**Conclusions:**

POU3F3 might positively regulate NSCLC cell proliferation, migration and invasion through downregulation of miR-30d-5p.

## Background

Lung cancer is one of the most frequently diagnosed malignancies and is also a leading cause of cancer-related deaths in many countries of the world including the United States and China [1, 2]. At present, the prevention and radical treatment of lung cancer is still a major task in public health. Surgical resection is the most common radical treatment for lung cancer patients [3], while the clinical application of surgery is limited by the high prevalence of cancer metastasis at the time of first diagnosis [4]. The development of chemotherapy and immunotherapy has significantly improved the survival of lung cancer patients at advanced stages, while the development of chemoresistance after long-term use of chemical drugs leads to poor long-term outcomes [5].

The development of lung cancer involves both internal and external factors, such as the regulation of non-coding RNAs (ncRNAs), which are a group of non-protein-coding RNAs, which are transcripts with essential roles in cancer biology [6]. NcRNAs are divided into different subgroups, including long non-coding RNAs (lncRNAs), which are composed of more than 200 nucleotides and are key players in cancer development [7]. LncRNAs encode no proteins but regulate gene expression at multiple levels to participate in human diseases including lung cancer [6, 7]. In effect, regulating the expression of certain lncRNAs has shown great potentials in the development of targeted anti-cancer therapies [6, 7]. Besides, lncRNAs can also achieve their functions through interactions with microRNAs (miRNAs) [8], which are also critical players in cancer biology [9]. Long noncoding RNA POU class 3 homeobox 3 (POU3F3) is upregulated in esophageal squamous-cell carcinomas, suggesting its involvement in this disease [10]. MicroRNA-30d-5p (miR-30d-5p) inhibits lung cancer by targeting cyclin E2 (CCNE2) [11]. In the present study, we demonstrated that POU3F3 enhanced cancer cell proliferation, migration and invasion by downregulating miR-30d-5p in non-small cell lung cancer (NSCLC, adenocarcinoma), which was a major subgroup of lung cancer.

## Methods

### Research subjects

A total of 80 patients with NSCLC (adenocarcinoma) were enrolled in Guangdong Provincial Hospital of Integrated Traditional Chinese and Western Medicine between May 2016 and May 2018. Inclusion criteria: 1) patients who received pathological examinations and malignant tumors were confirmed by 3 experienced pathologists; 2) patients with complete medical record; 3) patients were willing to participate. Exclusions criteria: 1) patients with benigned lung tumors; 2) patients with other severe diseases. All patients signed written informed consent. This study was approved by the Ethics Committee of Guangdong Provincial Hospital of Integrated Traditional Chinese and Western Medicine.

### Human specimens and cell lines

Biopsy was performed on all patients to collect tumor and adjacent healthy tissue specimens. The specimens were confirmed by 3 experienced pathologists. All in vitro experiments in this study were performed using NCI-H23 and NCI-H522 cell lines, which were purchased from American Type Culture Collection (ATCC, Manassas, VA, USA). ATCC-formulated RPMI-1640 medium supplemented with 10% fetal bovine serum (FBS) was used to cultivate both cell lines in an incubator at 37 °C with 5% CO_2_.

### Real-time quantitative PCR (qRT-PCR)

Total RNAs were extracted using Trizol reagent (Invitrogen, USA). Reverse transcription was performed using SuperScript IV Reverse Transcriptase (Thermo Fisher Scientific) and PCR reactions were prepared using the Applied Biosystems™ Power SYBR™ Green PCR Master Mix. To detect miR-30d-5p precursor, miRNAs were extracted using the mirVana miRNA Isolation Kit (Thermo Fisher Scientific). MystiCq® microRNA cDNA Synthesis Mix (Sigma-Aldrich, St. Louis, MO, USA) was used to synthesize cDNA, and miScript SYBR Green PCR Kit (QIAGEN) was used to prepare PCR reaction systems. Primers of lncRNA POU3F3, miR-30d-5p, as well as GAPDH and U6 endogenous controls were provided by Sangon (Shanghai, China). PCR reactions were performed on ABI PRISM 7500 qRT-PCR machine (Applied Biosystems, Rockford, IL, USA). Data normalizations were performed based on 2^-ΔΔCT^ method. Primer sequences were: 5′-AATCACTGCAATTGAAGGAAAAA-3′ (forward) and 5′-CCTTGTTTTCCAACCCTTAGACT-3′ (reverse) for POU3F3; 5′-GTCTCCTCTGACTTCAACAGCG-3′ (forward) and 5′-ACCACCCTGTTGCTGTAGCCAA-3′ (reverse) for GAPDH; 5′-GTTGTTTGTAAACAUCCCC-3′ (forward) and 5′-GTAGCAGCAAACATCTGACTG-3′ (reverse) for precursor miR-30d-5p. Forward primer of miR-30d-5p was 5′-TGTAAACATCCCCGACTGG-3′. Universal mature miRNA reverse primers and U6 primers were from the kit. The expression of pri-miR-30d-5p was determined by TaqMan™ Pri-miRNA Assay (Assay ID Hs03302839_pri; ThermoFisher).

### Cell transfections

POU3F3 gene (NCBI Accession: NR_037883.1) was inserted into pcDNA3.1 vector to establish POU3F3 expression vector. Vector construction service was provided by Sangon. Scrambled miRNA negative control 2 and hsa-miR-30d-5p miRNA Mimic were purchased from Sigma-Aldrich. Cell transfections were performed using Invitrogen™ Lipofectamine™ 2000. Transfection Reagent with 10 nM vectors and 40 nM siRNAs. Cells treated with Lipofectamine 2000 reagent only and cells transfected with empty vector were used as control and negative control cells, respectively. Cells were harvested at 24 h after transfection and overexpression of POU3F3 and miR-30d-5p were confirmed by qRT-PCR before subsequent experiments.

### Cell proliferation assay

Cell proliferation assay was performed at 24 h after transfection using the Cell Counting Kit 8 (ab228554, Abcam). ATCC-formulated RPMI-1640 medium containing 10% FBS was used to prepare single cell suspensions with cell concentration at 3 × 10^4^ cells/ml. Each well of a 96-well plate was filled with 0.1 ml cell suspension and cells were cultivated in an incubator at 37 °C with 5% CO_2_, followed by addition of 10 μl CCK-8 solution every 24 h until 96 h. Cells were then cultivated for an additional of 4 h, and optical density was measured at 450 nm.

### Transwell migration and invasion assay

In vitro*,* Transwell cell migration and invasion assays were performed to test cell migration and invasion abilities at 24 h after transfections. Cell suspensions (3 × 10^4^ cells/ml) were prepared using serum-free ATCC-formulated RPMI-1640 medium. Cell suspensions were transferred into upper Transwell chamber (0.1 ml/well), and the lower chamber was filled with ATCC-formulated RPMI-1640 medium containing 20% FBS. Cells were cultivated for 2 h, followed by staining of upper chamber membranes with 0.5% crystal violet (Sigma-Aldrich, USA) at room temperature for 20 min. An optical microscope was used to observe the stained cells. Matrigel (10%, 356,234, Millipore, USA) was used to coat upper chamber membrane for 6 h at 37 °C before invasion assay.

### Statistical analysis

There biological replicates were included in each experiment. Mean or mean ± standard deviation was used to express all data. Correlation analyses were performed by Pearson’s correlation coefficient. Comparisons between tumor tissues and adjacent healthy tissues were performed by paired t test. Comparisons among multiple cell groups were performed using ANOVA (one-way) and Tukey test. Chi-squared test was used to analyze the association between patients’ clinical data and expression levels of POU3F3 in NSCLC tissues. All statistical analyses were conducted using GraphPad Prism 6 software. *p* < 0.05 was considered to be statistically significant.

## Results

### POU3F3 and miR-30d-5p were dysregulated in NSCLC tissues

**Fig. 1:**
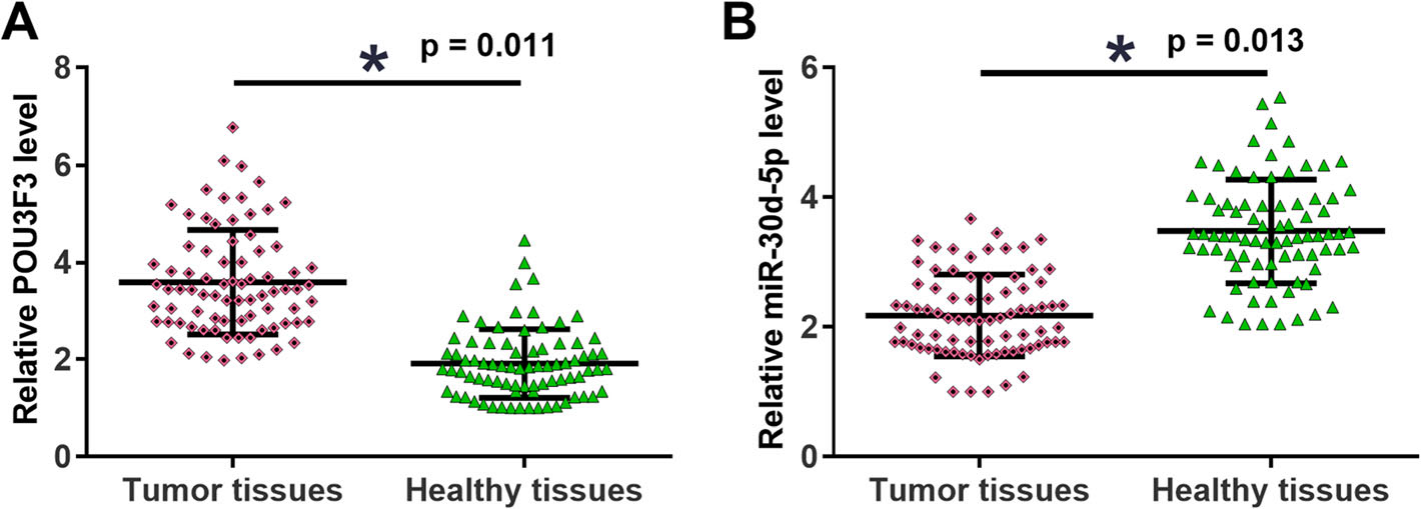
POU3F3 and miR-30d-5p showed opposite expression pattern in NSCLC. RT-qPCR was performed to explore the differential expression of POU3F3 and miR-30d-5p in tumor tissues of NSCLC patients. Compared with adjacent healthy tissues, POU3F3 was significantly upregulated (**a**), while miR-30d-5p was significantly downregulated (**b**) in tumor tissues (*, *p* < 0.05)

The results of qRT-PCR showed that, compared to adjacent healthy tissues, POU3F3 was significantly upregulated (Fig. 1a), while miR-30d-5p was significantly downregulated (Fig. 1b) in tumor tissues (*p* < 0.05). It is worth noting that expression levels of POU3F3 and miR-30a-5p were not significantly changed with the increasing of clinical stages (data not shown). Chi-squared test showed that expression levels of POU3F3 in NSCLC tissues were not significantly associated with patients’ gender, age, AJCC stages and smoking or drinking habits (Table 1, all *p* > 0.05).

**Table 1:** Association between patients’ clinical data and levels of POU3F3 expression

Items	Groups	Cases	High-expression (> 3.89)	Low-expression (< 3.89)	χ^2^	*p* value
Gender	Male	56	25	31	2.14	0.14
	Female	24	15	9		
Age	> 55 (years)	42	20	22	0.2	0.65
	< 55 (years)	38	20	18		
Stage	I	8	3	5	2.65	0.45
	II	19	12	7		
	III	33	17	16		
	IV	20	8	12		
Smoking	Yes	62	28	34	2.58	0.11
	No	18	12	6		
Alcohol consumption	Yes	54	24	30	0.25	0.15
	No	26	16	10		

### POU3F3 and miR-30d-5p were inversely correlated in tumor tissues

**Fig. 2:**
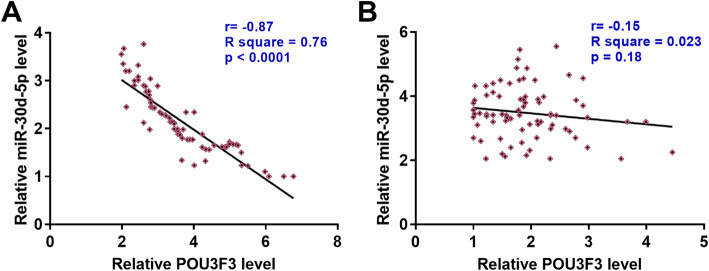
Expression levels of POU3F3 and miR-30d-5p were inversely correlated in tumor tissues. Pearson’s correlation coefficient analysis showed that expression levels of POU3F3 and miR-30d-5p were inversely correlation in tumor tissues (**a**), but not in adjacent healthy tissues (**b**)

Correlations between the expression levels of POU3F3 and miR-30d-5p were analyzed by Pearson’s correlation coefficient. POU3F3 and miR-30d-5p were significantly and inversely correlated across tumor tissues (Fig. 2a, r = − 0.87, *p* < 0.0001), but not in adjacent healthy tissues (Fig. 2b, r = − 0.15, *p* = 0.18).

### POU3F3 negatively regulated miR-30d-5p in NSCLC cell lines

**Fig. 3:**
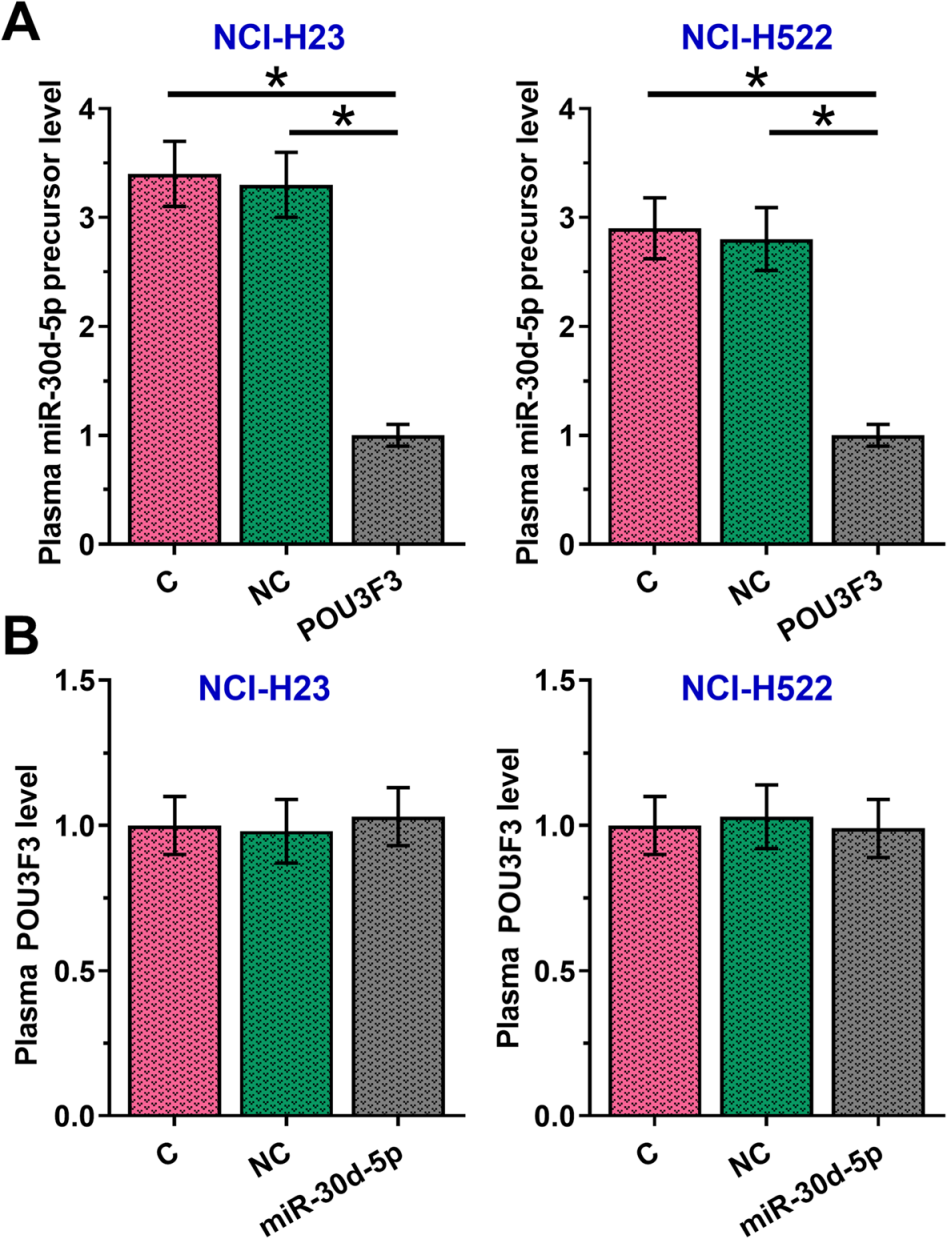
Overexpression of POU3F3 mediated miR-30d-5p inhibition in NSCLC cell lines. Overexpression of POU3F3 mediated miR-30d-5p inhibition in both NCI-H23 and NCI-H522 cell lines (**a**) (*, *p* < 0.05), while overexpression of miR-30d-5p did not affect the expression of POU3F3 (**b**)

To further investigate interactions between POU3F3 and miR-30d-5p, POU3F3 and miR-30d-5p were overexpressed in both NCI-H23 and NCI-H522 cell lines, followed by detecting the expression levels of POU3F3 and miR-30d-5p by RT-qPCR. As shown in Fig. 3a, overexpression of POU3F3 resulted in the downregulation of miR-30d-5p in both cell lines (Fig. 3a, *p* < 0.05), while overexpression of miR-30d-5p did not affect the expression of POU3F3 (Fig. 3b). It was worth noting that we also analyzed the expression of miR-30d-5p in cells transfected with POU3F3 overexpression, which also showed downregulated miR-30d-5p precursor and pri-miR-30d-5p (data not shown), indicating that POU3F3 downregulated miR-30d-5p at transcription level. In addition, RNA-RNA interaction predicted by IntaRNA 2.0 (http://rna.informatik.uni-freiburg.de/IntaRNA/Input.jsp) revealed no strong binding site of miR-30d-5p on POU3F3 (data not shown). Moreover, preliminary dual-luciferase activity assay (co-transfection of miR-30d-5p + POU3F3 or NC miRNA + POU3F3) showed that miR-30d-5p and POU3F3 may not directly interact with each other (data not shown).

### Overexpression of POU3F3 promoted cancer cell proliferation through miR-30d-5p

**Fig. 4:**
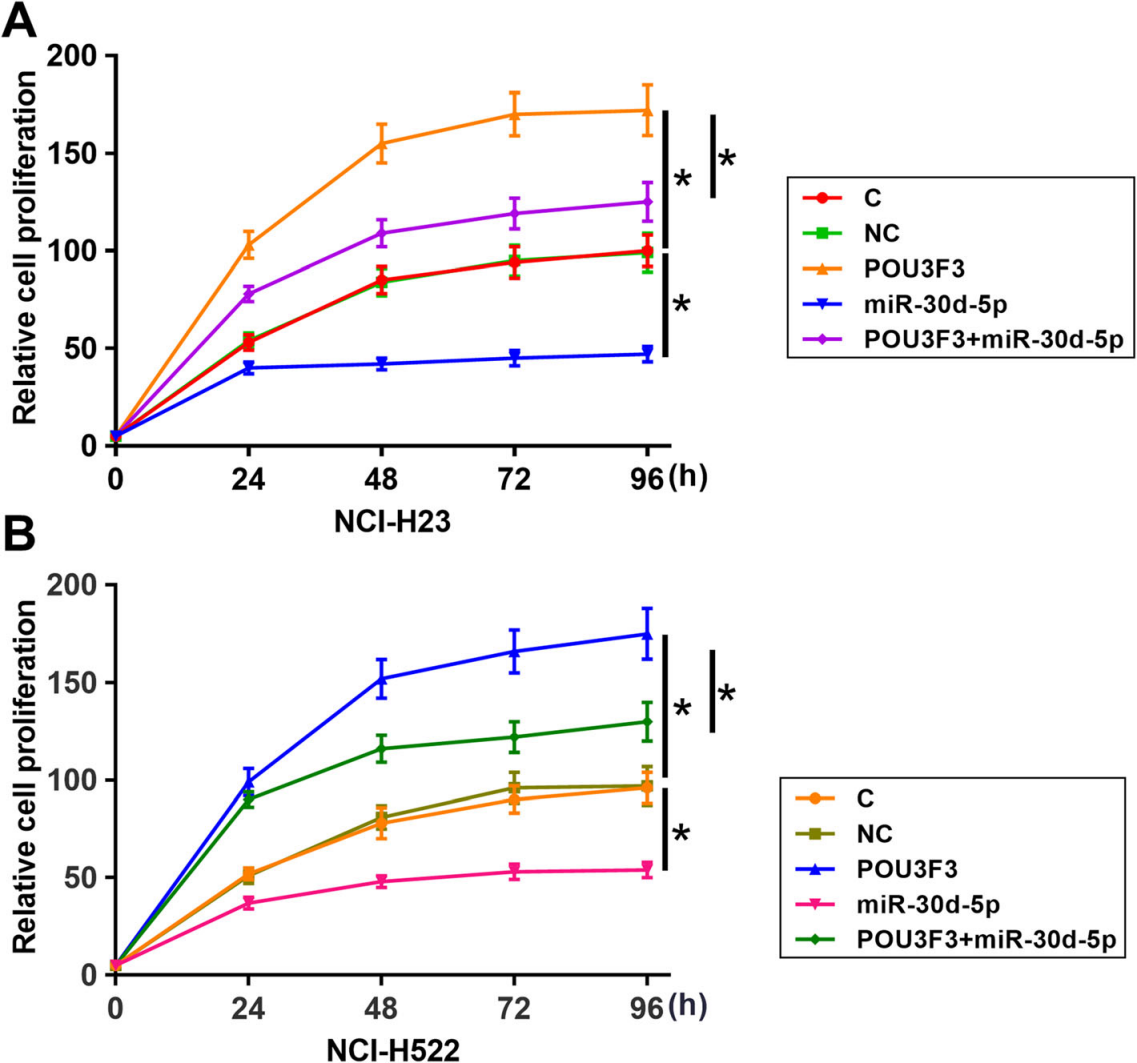
Overexpression of POU3F3 promoted cancer cell proliferation through miR-30d-5p. Overexpression of POU3F3 mediated enhanced, while overexpression of miR-30d-5p mediated inhibited proliferation of both NCI-H23 and NCI-H522 cell lines. In addition, overexpression of miR-30d-5p attenuated the effects of overexpressing POU3F3 on cancer cell proliferation (*, *p* < 0.05)

In vitro cell proliferation assay results showed that, in comparison to un-transfected cells (control, C) and cells transfected with empty vectors (negative control, NC), cells with the overexpression of POU3F3 showed enhanced proliferation ability. In contrast, cells with the overexpression of miR-30d-5p showed inhibited proliferation ability (Fig. 4, *p* < 0.05). In addition, in comparison to cells with the overexpression of POU3F3 only, cells with the overexpression of both POU3F3 and miR-30d-5p showed significantly reduced cell proliferation ability (*p* < 0.05).

### Overexpression of POU3F3 promoted cancer cell migration and invasion through miR-30d-5p

**Fig. 5:**
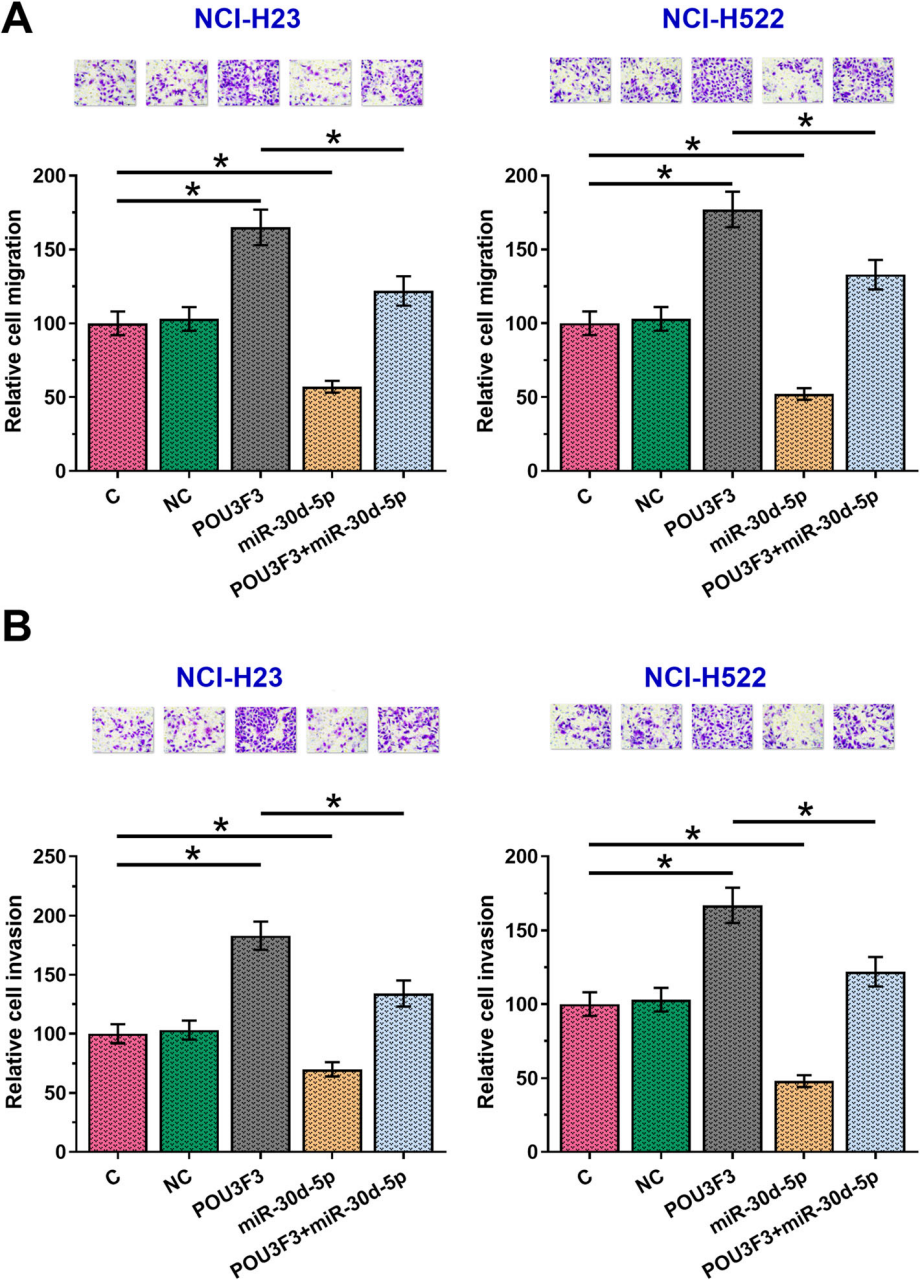
Overexpression of POU3F3 promoted cancer cell migration and invasion through miR-30d-5p. Overexpression of POU3F3 mediated enhanced, while overexpression of miR-30d-5p mediated inhibited migration (**a**) and invasion (**b**) of both NCI-H23 and NCI-H522 cell lines. In addition, overexpression of miR-30d-5p attenuated the effects of overexpressing POU3F3 on cancer cell migration and invasion (*, *p* < 0.05)

In vitro cell migration and invasion assay results showed that, compared with C and NC groups, cells with the overexpression of POU3F3 showed increased migration (Fig. 5a) and invasion (Fig. 5b) rates of both NCI-H23 and NCI-H522 cell lines (*p* < 0.05). In contrast, cells with the overexpression of miR-30d-5p showed decreased migration (Fig. 5a) and invasion (Fig. 5b) of both NCI-H23 and NCI-H522 cell lines (*p* < 0.05). In addition, compared with cells with the overexpression of POU3F3 only, cells with the overexpression of both POU3F3 and miR-30d-5p showed significantly inhibited cell migration and invasion ability (*p* < 0.05).

## Discussion

LncRNA POU3F3 is upregulated in esophageal squamous-cell carcinomas, indicating its involvement in cancer biology. The key finding of the present study is that POU3F3 is upregulated in NSCLC and overexpression of POU3F3 may promote NSCLC through the downregulation of miR-30d-5p.

MiR-30d-5p plays pivotal roles in different human diseases. Plasma levels of miR-30d-5p were upregulated in acute myocardial infarction patients and the overexpression of miR-30d-5p distinguished acute myocardial infarction patients from the healthy controls [12]. MiR-30d-5p also participated in hypoxic-ischemic injury by mediating cell autophagy and apoptosis [13]. In a recent study, Chen et al. reported that miR-30d-5p inhibited cancer cell proliferation and motility in NSCLC by targeting CCNE2 [11]. Consistent with previous studies, our results also showed inhibited proliferation, migration and invasion of NSCLC cells after the overexpression of miR-30d-5p, further confirming its role as a tumor suppressor in NSCLC.

Although miR-30d-5p participates in multiple human diseases, its functionality has not been well studied. MiR-30d-5p targets CCNE2, while its upstream regulators are still unknown. An increasing number of studies have shown that miRNAs can interact with lncRNAs to participate in cancer biology [14–16]. Our study focused on interactions between POU3F3 and miR-30d-5p in NSCLC, because our preliminary transcriptome analysis showed that expression levels of POU3F3 and miR-30d-5p were inversely correlated in tumor tissues of NSCLC patients. Our data support the hypothesis that POU3F3 is an upstream inhibitor of miR-30d-5p, and the inhibition of miR-30d-5p by POU3F3 is involved in the regulation of NSCLC cell proliferation, migration and invasion. Therefore, POU3F3/miR-30d-5p-CCNE2 signaling may be a novel signaling transduction pathway in NSCLC. It is also worth to note that the interaction between POU3F3 and miR-30d-5p is likely mediated by other pathological factors due to the lack of close correlation between POU3F3 and miR-30d-5p in healthy tissues. However, more studies are needed to identify those mediators.

Considering the fact that POU3F3 is upregulated in esophageal squamous-cell carcinomas [10], POU3F3 may also participate in lung squamous cell carcinoma. Our future studies will explore the role of POU3F3 in lung squamous cell carcinoma.

## Conclusions

In conclusion, POU3F3 plays an oncogenic role in NSCLC by downregulating tumor suppressor miR-30d-5p.

## Data Availability

The datasets used and analyzed during the current study are available from the corresponding author on reasonable request.

## Abbreviations

NSCLC: Non-small cell lung cancer
POU3F3: POU class 3 homeobox 3
ncRNAs: Non-coding RNAs
lncRNAs: Long non-coding RNAs
miRNAs: MicroRNAs
miR-30d-5p: MicroRNA-30d-5p
CCNE2: Cyclin E2
qRT-PCR: Real-time quantitative PCR
